# Right-to-Left Intra-cardiac Shunt in a COVID-19 Patient Leading to Stroke and Poor Prognosis: A Case Report and Review of the Literature

**DOI:** 10.7759/cureus.54421

**Published:** 2024-02-18

**Authors:** Ahmad Damlakhy, Husam Barham, Mohammad Omar, Zohaib Khan, Montaser Elkholy

**Affiliations:** 1 Internal Medicine, Detroit Medical Center/Sinai Grace Hospital/Wayne State University, Detroit, USA; 2 Internal Medicine, Balqa Applied University, Al-Salt, JOR

**Keywords:** ards (acute respiratory distress syndrome), pulmonary arterial hypertension, refractory hypoxemia, superimposed infections, paradoxical emboli, covid 19, intracardiac shunt

## Abstract

Coronavirus disease 2019 (COVID-19) often presents with a wide range of complications, including respiratory distress, acute respiratory distress syndrome (ARDS), and hypercoagulable states with resultant cerebrovascular incidents. Intra- and extra-pulmonological shunts can further complicate patient courses, leading to persistent hypoxemia and paradoxical emboli, resulting in potentially life-threatening consequences, necessitating a comprehensive, multidisciplinary approach to patient care. Here we present the case of a 73-year-old male who experienced severe persistent hypoxemic respiratory failure, superimposed methicillin-resistant *Staphylococcus aureus *(MRSA) pneumonia, and stroke with a previously undiagnosed patent foramen ovale (PFO) contributing to his clinical presentation.

## Introduction

Healthcare systems worldwide have faced unprecedented challenges due to the enduring coronavirus disease 2019 (COVID-19) pandemic. The complete understanding of hypoxemia in COVID-19 patients remains elusive, as it appears to stem from various pathological processes including changes in hypoxic pulmonary vasoconstriction (HPV), coagulopathy, and ventilation/perfusion (V/Q) mismatch. However, it is crucial to emphasize that even uncomplicated pneumonia induced by the virus can escalate to acute respiratory distress syndrome (ARDS) [1].

Although COVID-19 has gained recognition for its association with interstitial pneumonia and the development of ARDS, It has been implicated in a multitude of complications beyond its respiratory effects. Mounting evidence suggests that the virus exerts a direct influence on the various neurological symptoms that manifest throughout the course of the disease, as well as its ability to provoke a pro-thrombotic state, thereby increasing the risk of cerebrovascular events such as strokes. Stroke was demonstrated to be an infrequent, albeit potentially life-threatening, complication of COVID-19, affecting approximately 1-3% of hospitalized patients, and up to 6% of those in the intensive care unit (ICU) [2,3].

We hypothesize that, in select patients, some complications of COVID-19 may be due to right-to-left (RTL) shunt. Shunt may be intra-pulmonary or extra-pulmonary and can cause paradoxical embolization, hypoxia, and platypnea orthodeoxia. Shunt across patent foramen ovale (PFO) is reported in ARDS cases, often associated with poor response to positive expiratory pressure [4,5].

This case serves to assess the role of intracardiac or intrapulmonary shunt in COVID-ARDS-related hypoxemia, and their possible association with COVID-ARDS respiratory mechanics. Here, we present a COVID-19 patient who presented with refractory hypoxemia associated with previously undiagnosed PFO leading to stroke.

## Case presentation

A 73-year-old man, with a history of congestive heart failure (CHF), obstructive sleep apnea (OSA), chronic kidney disease (CKD) stage 3a, and hypertension presented to the emergency department (ED) experiencing worsening shortness of breath and a productive cough for four days, despite using four liters of oxygen at home. He denied fevers, chills, nausea, vomiting, or changes in urinary habits and mentioned that his sputum was white to gray. Notably, he had been discharged four days ago from another hospital, where he received oral steroids and home oxygen for COVID-19 pneumonia.

Upon examination, he was alert and oriented with a blood pressure of 139/75 and a heart rate of 105. Initially, his oxygen saturation was 76% on a 4 L nasal cannula, but it improved to 90% on 15 L via a nonrebreather face mask. The physical exam revealed decreased air entry bilaterally and fine crackles in the lower bases. Cardiac auscultation showed S1 and S2 with no additional heart sounds or edema. The patient demonstrated full strength and movements in all extremities with no focal neurological deficits.

**Figure 1:**
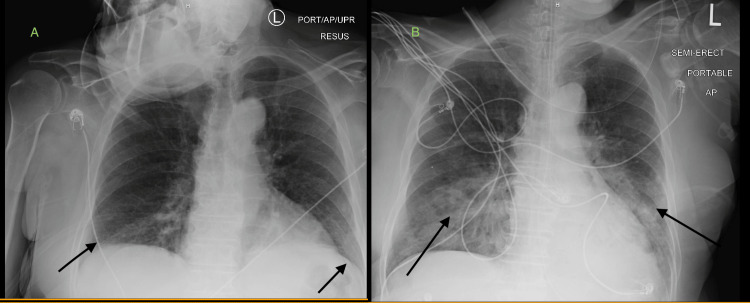
Chest X-ray images (1A) Black arrows show patchy opacity within the lung bases; (1B) Black arrows show bilateral mid and lower lung field airspace opacities.

Despite being on 15 L of oxygen via a nonrebreather mask, the patient remained hypoxemic in the ED. Arterial blood gas (ABG) test showed a pH of 7.43 (normal pH range 7.35-7.45), pCO_2_ 39 (normal range 35-45mmHg), pO_2_ 57 (normal range 80-100 mmHg), HCO_3_ 25 (normal range:22-28 meq/L), and O_2_ saturation 89% (normal range: 94-100%). Blood work-up indicates a notable leukocytosis with a white blood cell count of 28.1 K/CUMM (normal range 3.5-10.6 K/CUMM). Urinalysis was negative for infectious processes. Chest X-ray revealed bilateral basilar infiltrates (Figure 1A), computed tomography pulmonary angiogram (CTPE) ruled out pulmonary embolism, and brain computed tomography (CT) without contrast was unremarkable. Later on, the patient was intubated due to persistent hypoxemia and respiratory distress despite the use of bilevel-positive airway pressure (BiPAP). During hospitalization, the patient received empiric antibiotics for suspected infection and possible sepsis, including IV vancomycin and cefepime. Respiratory culture was positive for methicillin-resistant *Staphylococcus aureus* (MRSA), which led to a narrow antibiotic choice of vancomycin only. Repeat chest X-rays suggested ARDS due to pneumonia (Figure 1B).

**Figure 2:**
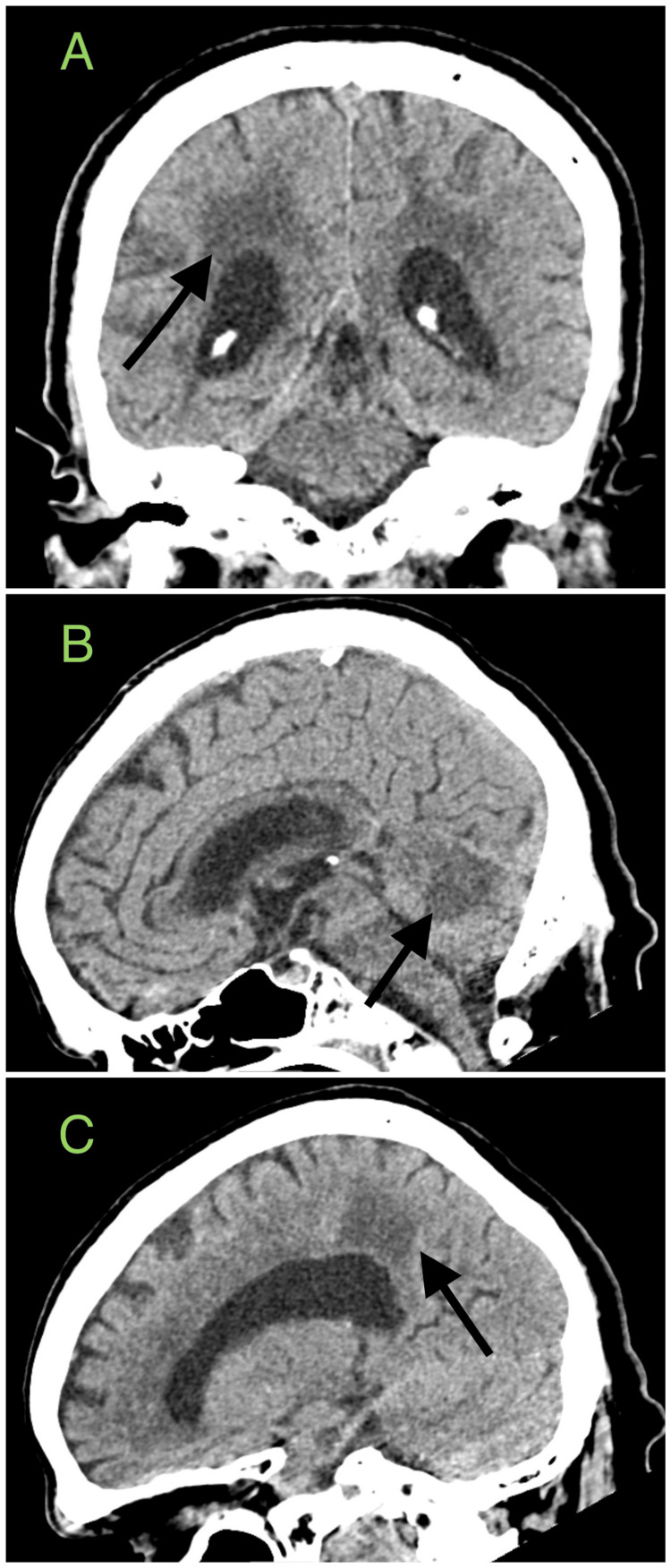
Brain CT without contrast Black arrows in images A, B, and C show hypoattenuation representing acute infarction.

Infectious disease (ID) consultation for COVID-19 with superimposed MRSA pneumonia was recommended against immunomodulated therapy but dexamethasone 6 mg IV daily was initiated. Two days later, he was weaned off sedation; however, he showed minimal improvement in the neurological exam, and a CT head without contrast revealed multiple infarctions, including a large acute to subacute non-hemorrhagic infarction in the superior cerebellar vermis, the pons, right middle cerebellar peduncle, and dorsal midbrain (Figure 2).

Further investigations with head and neck CT angiography showed multifocal atherosclerotic calcifications without significant stenosis. Brain MRI confirmed acute infarctions. A transthoracic echocardiogram revealed an ejection fraction of 60% with a positive agitated bubble study for PFO and right-to-left shunting. This was later confirmed by a transesophageal echocardiogram, which showed small PFO with RTL shunt with no intracardiac thrombus (Videos 1, 2).

**Video 1:** Transesophageal echocardiogram Color Doppler showed a patent foramen ovale (PFO) with a right-to-left shunt.

**Video 2:** Transesophageal echocardiogram A Bubble study showed a patent foramen ovale (PFO) with a right-to-left shunt.

Venous duplex of the upper and lower limbs showed no concerns for deep vein thrombosis. Cardiology was consulted with no plans for acute intervention due to the patient’s poor prognosis, and the patient was started on aspirin 81 mg daily, plavix 75 mg daily, and atorvastatin 80 mg daily.

Despite maximum ventilator settings, appropriate proning position trials, and a hospital stay of 19 days, the patient's PaO_2_ remained in the 60s-70s mmHg with no improvement. Daily physical exams indicated deteriorating neurological status. Unfortunately, after 19 days in the hospital, the patient passed away due to cardiopulmonary arrest, likely due to prolonged hypoxemia.

## Discussion

This case report describes a complex and challenging clinical scenario involving a 73-year-old man with multiple comorbidities, who presented with acute hypoxemic respiratory failure due to COVID-19 infection and superimposed MRSA pneumonia. The patient also experienced a stroke, further complicating the management of his condition.

The primary concern in this case was the respiratory compromise caused by COVID-19 infection. The patient required mechanical ventilation due to severe hypoxemia despite initial treatment with steroid therapy. The decision to intubate was based on the patient's deteriorating respiratory status and the failure of noninvasive ventilation to improve oxygenation. The identification of superimposed MRSA pneumonia necessitated the initiation of appropriate antibiotic therapy. This highlights the importance of considering bacterial coinfections in COVID-19 patients, as they can significantly impact disease severity and outcomes.

The patient's neurological complications, specifically the multifocal infarctions, further added to the complexity of the case. The infarction was identified through brain imaging, revealing ischemic changes. This finding raised concerns regarding the patient's neurological prognosis and emphasized the need for neurocritical care consultation. Dual antiplatelet therapy and high-dose statin were recommended to mitigate further cerebrovascular events and manage the infarction.

COVID-19 infection likely played a role in the development of the cerebrovascular disease in this patient. It has been associated with a hypercoagulable state, leading to an increased risk of thrombotic events, including cerebrovascular complications. The systemic inflammation and endothelial dysfunction caused by the virus can trigger a cascade of prothrombotic factors, potentially leading to ischemic and hemorrhagic strokes [6]. The presence of multifocal infarctions in the cerebellum, pons, basal ganglia, and internal capsule, along with atherosclerotic calcifications in the cerebral arteries, suggests a combination of COVID-19-induced hypercoagulability and pre-existing vascular risk factors as contributing factors to the cerebrovascular disease observed in this patient.

COVID-19 infection can elevate pulmonary vascular resistance, leading to reported cases of pulmonary arterial hypertension. Furthermore, a study involving 161 COVID-19 patients with ARDS revealed a 35% detection rate of RTL shunt. This occurrence may be attributed to COVID's-19 capacity to increase pulmonary vascular resistance, resulting in pulmonary arterial hypertension, heightened pressure on the right side of the heart, and the potential development of RTL shunt. The occurrence of RTL shunt between the atria further contributed to the patient's refractory hypoxemia. This finding, along with the persistent need for high oxygen requirements despite proning, suggests that the shunt was a significant cardiovascular finding. These comorbidities, combined with COVID-19 infection, create a complex clinical contributor to the patient's respiratory distress. The etiology and significance of this shunt in the context of COVID-19 infection require further investigation [7,8].

The patient's medical history of CHF, hypertension, OSA, and CKD likely contributed to his vulnerability to respiratory failure and stroke, which requires a multidisciplinary approach to ensure optimal management and outcomes. Close collaboration between infectious disease specialists, critical care physicians, cardiologists, and neurologists was essential in this case to address the various aspects of the patient's condition [9-12].

It is important to note that the management of COVID-19 infection has evolved over time, and the treatment strategies employed in this case report are based on the available evidence at the time of writing. As new research emerges and treatment guidelines are updated, the approach to managing COVID-19 and its complications may continue to evolve.

## Conclusions

This case report highlights the challenges faced in managing COVID-19 infection patients with multiple comorbidities and complications. The interplay between respiratory failure, superimposed bacterial pneumonia, cerebrovascular disease, intra-cardiac shunt, and COVID-19-induced hypercoagulability underscores the importance of a comprehensive and individualized treatment approach. Multidisciplinary collaboration, timely interventions, and close monitoring are crucial in optimizing outcomes for patients with similar presentations. Further research is needed to enhance our understanding of the pathophysiology and management of COVID-19 infection in the presence of an intracardiac shunt.
